# Factors affecting patient activation among patients with systemic lupus erythematosus

**DOI:** 10.1038/s41598-024-51827-9

**Published:** 2024-01-18

**Authors:** Zhixia Wang, Yuqing Song, Lihong Ou, Dengbin Liao, Lingxiao He, Qian Ning, Yanling Chen, Hong Chen

**Affiliations:** 1 Department of Orthopedic Surgery, West China Hospital, Sichuan University, No. 37 Guoxuexiang, Chengdu, 610041 Sichuan People’s Republic of China; 2 Trauma Center, West China Hospital, Sichuan University, No. 37 Guoxuexiang, Chengdu, 610041 Sichuan People’s Republic of China; 3 Present Address: Leshan Vocational and Technical College, No. 1336 Qingjiang Avenue, Leshan, 614000 Sichuan People’s Republic of China; 4 School of Nursing, Chengdu University of Traditional Chinese Medicine, No. 1166 Liutai Avenue, Chengdu, 610041 Sichuan People’s Republic of China; 5 Edson College of Nursing and Health Innovation, Arizona State University, 550 N 3rd Street, Phoenix, AZ 85004 USA; 6 Department of Rheumatology and Immunology, West China Hospital, Sichuan University, No. 37 Guoxuexiang, Chengdu, 610041 Sichuan People’s Republic of China; 7 West China School of Nursing/West China Hospital, Sichuan University, No. 37 Guoxuexiang, Chengdu, 610041 Sichuan People’s Republic of China

**Keywords:** Rheumatic diseases, Health care, Health services

## Abstract

There are limited published studies on patient activation among patients with systemic lupus erythematosus (SLE) in China. Disease activity can significantly influence a patient's perception of their condition, subsequently impacting patient activation. However, the mechanisms through which disease activity influences patient activation remain poorly understood. This study aimed to investigate patient activation among patients with SLE in China and explore the influencing factors. We conducted a cross-sectional study from June to December 2021 at a rheumatology and immunology department of a tertiary hospital in Chengdu, China. Data were collected by questionnaire, including general information, disease activity, quality of chronic illness care, health literacy, self-efficacy, motivation, social support, and patient activation. A patient activation model was constructed based on the conceptual framework derived from the individual and family self-management theory. To evaluate the moderating effect of disease activity on patient activation model, participants were divided into two subgroups (low disease activity group and high disease activity group). 426 SLE patients were included. The mean score of patient activation among SLE patients was 63.28 ± 11.82, indicating that most SLE patients lacked skills and confidence to stick with health-promoting behaviors. Health literacy, social support, and self-efficacy had the greatest effect on patient activation. In the multi-group analysis, social support and health literacy contributed more to patient activation in SLE patients with high and low disease activity, respectively. Patient activation among SLE patients in China was at the third level. Healthcare professionals should help them adhere to health-promoting behaviors. Health literacy, social support, and self-efficacy are vital factors for patient activation. These factors should be prioritized based on disease activity when developing individually tailored interventions for patient activation.

## Introduction

Systemic lupus erythematosus (SLE) is a systemic autoimmune disease^1^. It’s characterized by abundant autoantibodies, high heterogeneity, recurrent relapses, and multisystem or multiorgan involvement^1,2^. The prevalence of SLE is approximately 0–241 cases per 100,000 persons in the world^3^. In China, the prevalence is 30–100/100,000, ranking second worldwide^2,4^. There was an estimate of more than one million SLE patients in China^5^. The persistent nature, frequent flare, and elevated risk of comorbidities contribute to an annual medical cost of $33,369 per patient^6^. Approximately 5.2% of SLE patients bore direct medical costs that exceeded annual household income^7^. Indirect costs were 2.33 times higher than direct costs^6,7^. Thus, SLE has caused a large burden to patients, their families, and society.

SLE patients are suggested to manage their conditions actively^2^. They should follow therapeutic regimen and engage in non-pharmacological interventions^2^. However, previous studies have identified suboptimal medication adherence with a rate of under 60% and physical inactivity among SLE patients, etc.^8,9^. Thus, healthcare providers should take measures to improve self-management among SLE patients.

Patient activation is a reliable predictor for self-management and a reference for self-management interventions^10^. The definition of patient activation is that patients believe their important role in self-managing care, cooperating with healthcare providers, and maintaining health together with function and have the skills and behavioral styles to achieve these responsibilities and to access high-quality health care^10^. People with higher patient activation engage more in health-promoting behaviors with resultant better health outcomes^11^. Patient activation can also affect patients’ future clinical indicators^12^. In addition, patients with better activation had lower healthcare expenditure and readmission rates^13^. Consequently, efforts should be made to promote patient activation among SLE patients. However, there is a paucity of research on patient activation among SLE patients in China^14,15^.

Previous studies revealed that patient activation is affected by disease-related, sociodemographic, and psychosocial factors^14^. However, it is little known about how these factors impact patient activation. The lack of research on patient activation and pathways of influencing factors restricts development of effective and customized interventions. Thus, the purpose of this study was to investigate patient activation among SLE patients in China and analyze the pathways of contributing factors to patient activation.

**Figure 1:**
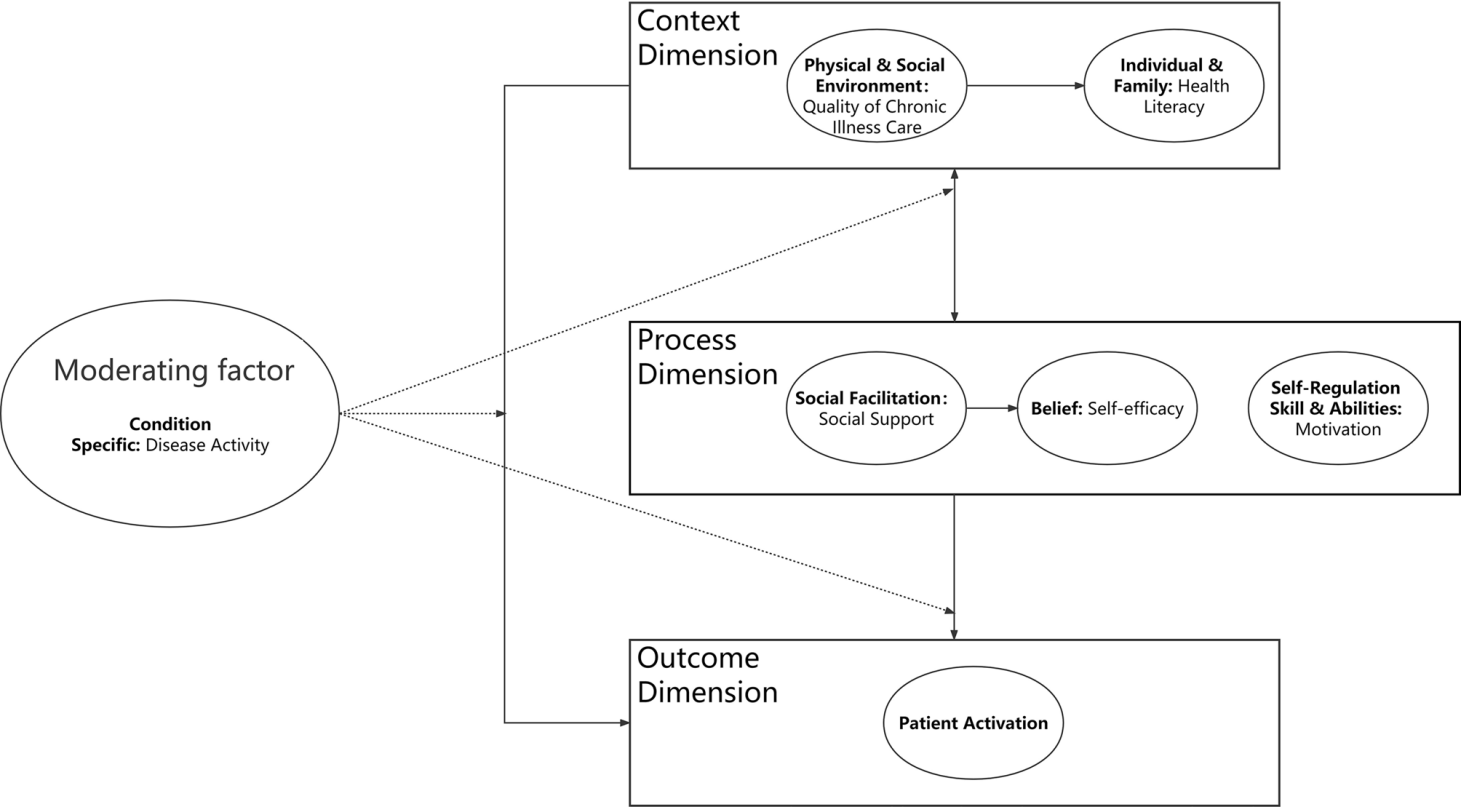
The conceptual framework for the patient activation model in people with systemic lupus erythematosus.

Patient activation is initially used as a predictor of self-management^11^. It is now considered equivalent to self-management^16^. The Individual and Family Self-Management Theory (IFSMT) is a widely used theory to explain self-management^17^. IFSMT comprises the context, process, and outcome dimensions^17^. The conceptual framework of this study was developed based on IFSMT and previous studies (Fig. 1)^14,17^.

Previous research demonstrated that disease activity, quality of chronic illness care, and health literacy are important factors affecting patient activation in the context dimension^14,17^. Disease activity is vital for reflecting the severity of disease, therapeutic effect, and risk of organ damage^2^. It can be regarded as a contributing factor since the way patients cope with conditions depends on their perceptions and beliefs about the severity of disease^14,17,18^. There are numerous benefits of evaluating the effects of antecedent factors on patient activation under different conditions. However, previous studies have not explored the effect of influencing factors on patient activation in SLE patients across different levels of disease activity. Therefore, disease activity was explored as a moderating variable that may affect the patient activation model in this study.

In the process dimension, self-efficacy, motivation (including autonomous motivation, introjected regulation, external regulation, and amotivation), and social support are crucial variables for patient activation. According to IFSMT^17^, patient activation is directly affected by variables in the process dimension. It can be directly and indirectly impacted by elements in the context dimension. Besides, social support has a positive effect on health literacy and interacts with quality of chronic illness care^19,20^. Thus, some variables in the context and process dimension might interact with each other. The hypotheses of this study are as follows: (1) quality of chronic illness care, health literacy, self-efficacy, social support, and the other three types of motivation, except for amotivation, positively impact patient activation; (2) disease activity has a moderating effect on patient activation model (Fig. 1). Thus, this study aimed to investigate patient activation among SLE patients in China and analyze the pathways of contributing factors to patient activation based on IFSMT.

## Methods

### Study design and participants

This is a cross-sectional study conducted in the rheumatology and immunology department of a tertiary hospital in Chengdu, China from June to December 2021. Inclusion criteria for participants were: (1) diagnosed with SLE^21^; (2) 18 years of age and above; (3) able to read and (or) communicate in Chinese; and (4) willing to participate in this study. Exclusion criteria were: (1) suffering from severe complications, such as lupus encephalopathy, lupus nephritis, or infection; (2) medical history of serious neurological diseases, psychiatric disorders, or cognitive dysfunction; (3) pregnant female patients; and (4) suffering from serious comorbidities that might affect their participation in healthcare.

According to the sample size calculation formula for the study of influencing factors n = 4U_α_^2^S^2^/δ^2^^22^ and the result of Fortin et al.^14^, α = 0.05 and S = 13.5 were selected. The tolerance error was set at 0.25 times the standard deviation^23^, and the sample size for this study should be at least 308 cases given 20% invalid questionnaires. Finally, 426 SLE patients were recruited by convenience sampling.

### Data collection

Disease activity was calculated based on participants’ electronic medical records. A self-report questionnaire was used to assess the disease-related characteristics, sociodemographic information, patient activation, quality of chronic illness care, health literacy, social support, self-efficacy, and motivation of participants. The participants independently completed the printed questionnaires. If participants had problems reading or writing the questionnaire, the investigator provided assistance with completion. The investigator would check questionnaires after the participants had finished.

### Measures

The self-report questionnaire measured patients’ general information, patient activation, quality of chronic illness care, health literacy, self-efficacy, motivation, and social support.

### General information

The general information questionnaire included socio-demographic and disease-related characteristics. Socio-demographic information consisted of age, gender, ethnic group, marital status, educational level, religion, region of residence, monthly household income per capita, drinking status, and smoking status. Disease-related information included disease duration and age of onset.

### Patient activation

Patient activation was assessed by the Chinese version of 13-item Patient Activation Measure (PAM-13)^24^. It’s a scale testing patients' knowledge, skills, actions, and beliefs about participation in healthcare. Each item is rated on a 5-point scale from 1 (strongly disagree) to 4 (strongly agree) and 0 for inapplicability. The standardized total score ranges from 0 to 100. A higher score suggests better activation. PAM-13 is divided into 4 levels: (1) patients in level 1 (≤ 47.0) are passive recipients of care and unaware of their crucial role in health management; (2) patients of level 2 (47.1–55.1) recognize their important role in disease management but lack the confidence and knowledge to take action; (3) patients of level 3 (55.2–67.0) have started to take actions recommended by healthcare professionals while without the confidence and skills to persist; (4) patients of level 4 (≥ 67.1) actively engage in various health-promoting behaviors consistently, while additional assistance is required when facing stress or new health crises^10^. Patient activation can also be categorized as low activation (levels 1 and 2) and high activation (levels 3 and 4)^25^. The internal consistency (Cronbach’s alpha = 0.82) and content validity (0.87) of PAM-13 were satisfactory^24^. The Cronbach’s alpha was 0.824 in this study.

### Disease activity

Disease activity was evaluated by the Systemic Lupus Erythematosus Disease Activity Index 2000 (SLEDAI-2K)^2,26^, which assesses the patient’s status in the 10 days preceding a visit. SLEDAI-2K assesses 24 indicators for 9 systems or organs, such as nervous system damage, vascular impairment, renal insufficiency, etc. The total score is 0–105 and higher score indicates higher disease activity. SLEDAI-2K is widely used for its ease of application and evaluation of emerging signs or symptoms.

### Quality of chronic illness care

The quality of chronic illness care was measured by the Chinese version of Patient Assessment of Chronic Illness Care (PACIC)^27^. PACIC is a 20-item scale testing patient initiative, delivery system design, goal setting, problem-solving/continuity, and follow-up/coordination. The total score is the mean of score for each entry (possible range = 1–5). A higher score suggests superior quality of chronic illness care. PACIC is a reliable and valid tool^28^. The Cronbach’s alpha of PACIC and subscales were 0.933 and 0.724–0.871 in this study.

### Health literacy

Health literacy was measured using the Chinese version of the Health Literacy Management Scale (HeLMS)^29^. HeLMS includes 4 dimensions with 24 entries (possible range = 1–5). It assesses the ability of information acquisition and communication as well as willingness for health improvement and economic support. The total score ranges from 24 to 120 with a higher score indicating greater health literacy. The Chinese version of HeLMS is a reliable and valid instrument^29^. The Cronbach’s alpha of HeLMS and the four dimensions were 0.864 and 0.752–0.979 in our sample.

### Self-efficacy

Self-efficacy was assessed by the Chinese version of 6-item Self-efficacy for Managing Chronic Disease (SEMCD)^30^. The total score is the mean of all the entries (possible range = 1–10). Higher scores represent greater confidence in managing conditions. The internal consistency, retest reliability, and criterion validity were 0.96, 0.98, and 0.373–0.607^30^, with an acceptable Cronbach’s alpha of 0.784 in this study.

### Motivation

The motivation was evaluated by the Chinese version of the Treatment Self-regulation Questionnaire (TSRQ)^31^. TSRQ is a 15-item Likert scale-based questionnaire (possible range = 1–15). TSRQ tests four sub-dimensions including autonomous motivation, introjected regulation, external regulation, and amotivation. A higher score in the subscale indicates stronger motivation in that form. Previous studies revealed good validity and reliability of the TSRQ^31,32^. The subscales’ Cronbach’s alpha were 0.856, 0.643, 0.663 and 0.506 in this research^32^.

### Social support

Social support was assessed using the Chinese version of Perceived Social Support Scale (PSSS)^33^. PSSS is a 12-item Likert scale questionnaire with an item score of 1–7. The total score ranges from 12 to 84. A higher score indicates superior support. The Cronbach's alpha fo PSSS was 0.922, and the factor loading coefficients of the twelve items were generally above 0.5^33^. The Cronbach's alpha was 0.903 in the current study.

### Statistical analysis

Descriptive statistics, correlation analysis, and internal consistency reliability were analyzed via SPSS 25.0 software (IBM Corp., Armonk, NY, USA). Path analysis is a special form of structural equation modeling. It was performed to test the conceptual framework with SPSS Amos 24.0 software (an extension of SPSS, IBM Corp., Armonk, NY, USA). A range of model fit indicators were used to evaluate the model fit: χ^2^/degree of freedom (expected χ^2^/df < 2, *p* value > 0.05), comparative fit index (CFI; expected value > 0.90), goodness of fit Index (GFI; expected value > 0.90), adjusted goodness of fit index (AGFI; expected value > 0.90), root mean square error of approximation (RMSEA; expected value < 0.08), standardized root mean square residual (SRMR; expected value < 0.08), normed fit index (NFI; expected value > 0.90), incremental fit index (IFI; expected value > 0.90), relative fit index (RFI; expected value > 0.90), and Tucker and Lewis index (TLI; expected value > 0.90)^34^. Standardized estimate (*β*), unstandardized estimate (B), critical ratio (CR), and *p* value were used to confirm the significance of the estimated coefficients for the hypothetical model. The bias-corrected bootstrap test was used to test the significance of each effect in the model^35^. The chain mediating effects in the model were tested by user-defined estimands and bias-corrected bootstrap test^35^.

After validating the patient activation model, disease activity was classified into low disease activity group and high disease activity group according to K-means cluster analysis^36^. Then, a multi-group analysis of the patient activation model was conducted^37^. The multi-group analysis was to examine if there were differences in the path coefficients among research variables between the two groups according to disease activity^37^. In multi-group analysis, the path coefficients in the constrained models were imposed to be equal. And data for the two groups had been analyzed simultaneously. The critical ratios for differences (CRD), difference in χ^2^ values between models (Δχ^2^), and* p* value were used to verify the significance of pathways difference between the two groups^38^.

### Ethics approval and consent to participate

This study was approved by the Ethics Committee on Biomedical Research, West China Hospital of Sichuan University (ID: 2021182). This study followed the Declaration of Helsinki and written informed consent was obtained from all participants before the start of the study.

## Results

### Sample

A total of 442 questionnaires were distributed and 16 were excluded for incomplete filling or continuous selection of the same answer. The analytic sample including 426 SLE patients is described in Table 1. The median, first quartile, and third quartile of SLEDAI-2K were 6.00 (2.00, 10.00), ranging from 0 to 21. The disease duration was 80.00 (27.00, 155.00) months, with a range of 0.00–454.00. As for patient activation, the mean score of PAM-13 was 63.28 ± 11.82, which was at level 3. Besides, 8.0%, 18.1%, 45.1%, and 28.9% of participants were at levels 1, 2, 3, and 4, respectively.

**Table 1:** General characteristics of the participants (n = 426).

Characteristics	n	%	Range
Gender			
Male	32	7.5	
Female	394	92.5	
Ethnic group			
Ethnic Han	396	93.0	
Ethnic minorities	30	7.0	
Marital status			
Single	97	22.8	
Married or on cohabitation	305	71.6	
Separation or divorced or widowed	24	5.6	
Educational level			
Primary school or below	45	10.6	
Middle school	108	25.4	
High school or technical secondary school	74	17.4	
College and university	199	46.7	
Religion			
No	389	91.3	
Yes	37	8.7	
Region of residence			
Urban	322	75.6	
Rural	104	24.4	
Monthly household income per capita(USD)			
< 145	47	11.0	
145–435	88	20.7	
435–724	128	30.0	
724–1449	99	23.2	
1449–4347	51	12.0	
> 4347	13	3.1	
Drinking status			
Never	355	83.3	
Drinking	50	11.7	
Abstinence	21	4.9	
Smoking status			
Never	394	92.5	
Smoking	23	5.4	
Abstinence	9	2.1	
Age of onset (year)			
< 18	63	14.8	
18–30	187	43.9	
31–40	112	26.3	
> 40	64	15.0	
Age (year, mean ± SD)	36.86 ± 10.94		18.00–70.00
Patient activation (mean ± SD)	63.28 ± 11.82		29.30–100.00
Quality of chronic illness care (mean ± SD)	3.03 ± 0.67		1.45–4.90
Health literacy (mean ± SD)	101.50 ± 8.76		66.00–118.00
Self-efficacy (mean ± SD)	7.20 ± 1.56		2.00–10.00
Autonomous motivation (mean ± SD)	38.56 ± 4.56		14.00–42.00
Introjected regulation (mean ± SD)	10.21 ± 3.37		2.00–14.00
External regulation (mean ± SD)	17.19 ± 6.07		4.00–28.00
Amotivation (mean ± SD)	6.65 ± 2.52		3.00–17.00
Social support (mean ± SD)	65.39 ± 11.20		30.00–84.00

*USD* USA dollar.

### Bivariate analysis

Table 2 shows the results of Pearson’s and Spearman’s correlation analysis. Patient activation was positively correlated with social support, quality of chronic illness care, health literacy, autonomous motivation together with self-efficacy. It was negatively correlated with amotivation and disease activity. Most of the correlations were weak. The relationships between patient activation and introjected regulation as well as external regulation were not significant.

**Table 2:** Correlations of continuous variables (n = 426).

	Social support	Quality of chronic illness care	Health literacy	Self-efficacy	Autonomous motivation	Introjected regulation	External regulation	Amotivation	Disease activity	Patient activation
Social support	1	0.215**	0.375**	0.236**	0.146**	0.167**	0.066	− 0.119*	− 0.051^△^	0.261**
Quality of chronic illness care	0.215**	1	0.239**	0.164**	0.075	0.132**	0.139**	0.007	0.029^△^	0.160**
Health literacy	0.375**	0.239**	1	0.311**	0.241**	0.174**	0.027	− 0.232**	− 0.100*^△^	0.375**
Self-efficacy	0.236**	0.164**	0.311**	1	0.301**	0.064	− 0.019	− 0.163**	0.011^△^	0.290**
Autonomous motivation	0.146**	0.075	0.241**	0.301**	1	0.296**	0.280**	− 0.051	− 0.030^△^	0.270**
Introjected regulation	0.167**	0.132**	0.174**	0.064	0.296	1	0.348**	0.025	0.008^△^	0.090
External regulation	0.066	0.139**	0.027	− 0.019	0.280**	0.348**	1	0.359**	− 0.017^△^	0.024
Amotivation	− 0.119*	0.007	− 0.232**	− 0.163**	− 0.051	0.025	0.359**	1	0.035^△^	− 0.203**
Disease activity	− 0.049	0.024	− 0.094	0.004	− 0.071	0.017	− 0.003	0.036	1^△^	− 0.130**
Patient activation	0.261**	0.160**	0.375**	0.290**	0.270**	0.090	0.024	− 0.203**	− 0.133**^△^	1

*p < 0.05, **p < 0.01; ^△^Spearman rank correlation.

### Hypothetical patient activation model test

**Figure 2:**
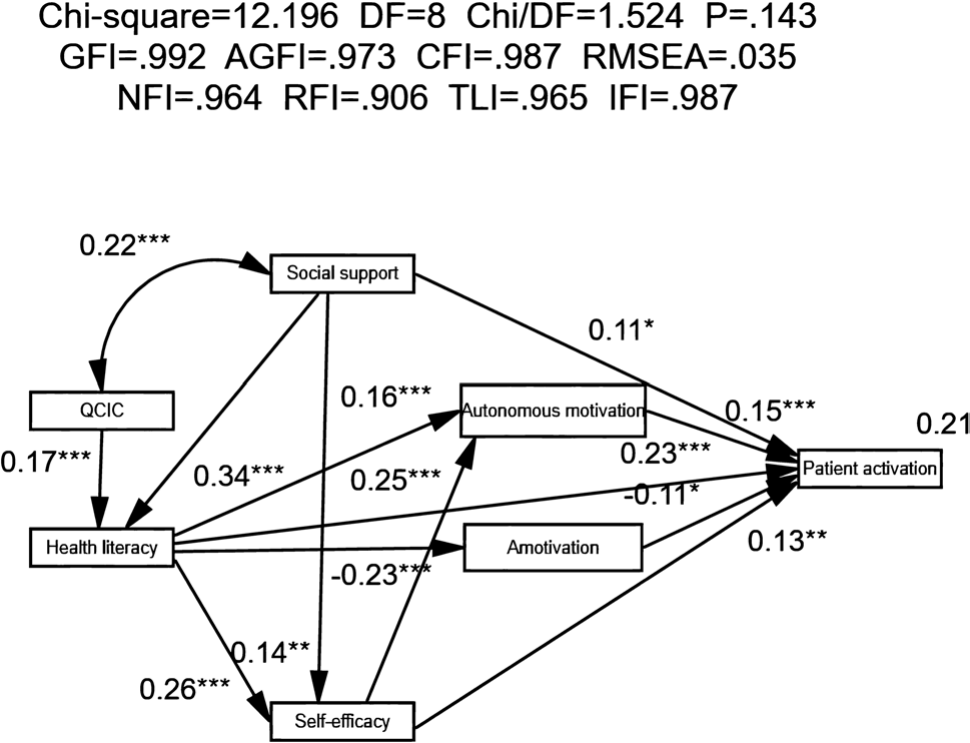
The model of patient activation among patients with systemic lupus erythematosus (with standardized regression coefficients). QCIC, quality of chronic illness care; *p < 0.05, *p < 0.01, ***p < 0.001.

The goodness of fit of the structural model based on the conceptual framework of research variables was generally good (χ^2^/df = 1.524, *p* = 0.143, CFI = 0.987, GFI = 0.992, AGFI = 0.973, RMSEA = 0.035, SRMR = 0.029, NFI = 0.964, IFI = 0.987, RFI = 0.906, TLI = 0.965) (Fig. 2).

### Estimates of the hypothetical patient activation model

The maximum likelihood method was used to estimate the parameter of the hypothetical model. The results of hypothetical model were presented with B, *β*, CR, and *p* values. As is shown in Table 3, all of the paths (13 paths) were statistically significant. Quality of chronic illness care, health literacy, self-efficacy, autonomous motivation, and social support had a positive effect on patient activation directly and (or) indirectly. Amotivation had a direct negative effect on patient activation. The result indicated that the support for hypothesis 1 was partial.

**Table 3:** Parameter estimates of variables for patient activation model (n = 426).

Path			*β*	B	S.E	C.R	*p*
Health literacy	⇠	Social support	0.339	0.265	0.035	7.486	< 0.001
Health literacy	⇠	Quality of chronic illness care	0.166	2.168	0.591	3.667	< 0.001
Self-efficacy	⇠	Health literacy	0.259	0.046	0.009	5.258	< 0.001
Self-efficacy	⇠	Social support	0.139	0.019	0.007	2.814	0.005
Autonomous motivation	⇠	Health literacy	0.163	0.085	0.025	3.403	< 0.001
Amotivation	⇠	Health literacy	− 0.232	− 0.067	0.014	− 4.907	< 0.001
Autonomous motivation	⇠	Self-efficacy	0.250	0.728	0.140	5.201	< 0.001
Patient activation	⇠	Autonomous motivation	0.154	0.399	0.119	3.363	< 0.001
Patient activation	⇠	Amotivation	− 0.108	− 0.506	0.208	− 2.429	0.015
Patient activation	⇠	Social support	0.108	0.114	0.049	2.302	0.021
Patient activation	⇠	Health literacy	0.233	0.314	0.066	4.736	< 0.001
Patient activation	⇠	Self-efficacy	0.129	0.974	0.357	2.728	0.006
Social support	⇠	Quality of chronic illness care	0.215	1.612	0.373	4.326	< 0.001

*β* standard estimate, *B* unstandardized coefficients, *S.E*. standard error, *C.R*. critical ratios.

### Effect analysis of the patient activation model

The bias-corrected bootstrap test was used to test the significance of each effect in the model. The results showed that the bootstrap 95% confidence interval (CI) for direct, indirect, and total effects of each exogenous variable did not overlap with zero, indicating that all effects were significant (Table 4). Health literacy, social support, and self-efficacy had the highest total effects on patient activation. Among them, health literacy and self-efficacy had greater direct effects than indirect effects. Social support contributed more to patient activation through indirect effect.

**Table 4:** Standard direct, indirect and total effect of the patient activation model (n = 426).

Endogenous variable	Exogenous variable	Total effect			Direct effect			Indirect effect			Total effect ranking
		*β*	Bootstrap 95%CI		*β*	Bootstrap 95%CI		*β*	Bootstrap 95%CI		
			Lower Bounds	upper Bounds		Lower Bounds	upper Bounds		Lower Bounds	upper Bounds	
Patient activation	Quality of chronic illness care	0.054**	0.021	0.093	–	–	–	0.054**	0.021	0.093	6
	Social support	0.242***	0.145	0.330	0.108*	0.005	0.205	0.134***	0.090	0.188	2
	Health literacy	0.327***	0.238	0.413	0.233***	0.142	0.321	0.094***	0.057	0.141	1
	Self-efficacy	0.167**	0.076	0.254	0.129**	0.038	0.220	0.038***	0.018	0.072	3
	Amotivation	− 0.108*	− 0.199	− 0.013	− 0.108*	− 0.199	− 0.013	–	–	–	5
	Autonomous motivation	0.154**	0.064	0.239	0.154**	0.064	0.239	–	–	–	4
Health literacy	Quality of chronic illness care	0.166**	0.068	0.255	0.166**	0.068	0.255	–	–	–	2
	Social support	0.339**	0.245	0.425	0.339**	0.068	0.425	–	–	–	1
Self-efficacy	Quality of chronic illness care	0.043**	0.015	0.083	–	–	–	0.043**	0.015	0.083	3
	Social support	0.227***	0.129	0.323	0.139**	0.040	0.239	0.088***	0.051	0.138	2
	Health literacy	0.259***	0.158	0.361	0.259***	0.158	0.361	–	–	–	1
Amotivation	Quality of chronic illness care	− 0.038**	− 0.071	− 0.018	–	–	–	− 0.038**	− 0.071	− 0.018	3
	Social support	− 0.079***	− 0.129	− 0.036	–	–	–	− 0.079***	− 0.129	− 0.036	2
	Health literacy	− 0.232***	− 0.344	− 0.114	− 0.232***	− 0.344	− 0.114	–	–	–	1
Autonomous motivation	Quality of chronic illness care	0.038**	0.015	0.068	–	–	–	0.038**	0.015	0.068	4
	Social support	0.112***	0.071	0.163	–	–	–	0.112***	0.071	0.163	3
	Health literacy	0.228***	0.141	0.311	0.163**	0.075	0.254	0.065***	0.031	0.115	2
	Self-efficacy	0.250***	0.136	0.366	0.250***	0.136	0.366	–	–	–	1

*p < 0.05, *p < 0.01, ***p < 0.001.

According to Hayes^39^, the significance of total effect or direct effect does not necessarily claim that of indirect effect. To identify the significance of chain mediating effects, user-defined estimands and the bias-corrected bootstrap test were executed. The bootstrap 95% CIs excluded 0 for each chain mediating effect (Table 5). The results indicated that all chain mediating effects were statistically significant. Health literacy, social support and quality of chronic illness care could positively influence patient activation through mediating variables.

**Table 5:** The chain mediating effects for the patient activation model in patients with systemic lupus erythematosus (n = 426).

Exogenous variable	Endogenous variable	Pathways	Estimates	S.E	Bootstrap 95%CI		*p*
					Lower bounds	Upper bounds	
Social support	Patient activation	Social support → Health literacy → Patient activation	0.083	0.022	0.047	0.132	< 0.001
		Social support → Health literacy → Autonomous motivation → Patient activation	0.009	0.004	0.003	0.020	0.001
		Social support → Health literacy → Amotivation → Patient activation	0.009	0.005	0.002	0.022	0.013
		Social support → Health literacy → Self-efficacy → Patient activation	0.012	0.005	0.004	0.025	0.005
		Social support → Health literacy → Self-efficacy → Autonomous motivation → Patient activation	0.004	0.002	0.001	0.008	< 0.001
		Social support → Self-efficacy → Patient activation	0.019	0.010	0.004	0.045	0.006
		Social support → Self-efficacy → Autonomous motivation → Patient activation	0.006	0.003	0.002	0.014	0.002
Quality of chronic illness care	Patient activation	Quality of chronic illness care → Health literacy → Patient activation	0.680	0.267	0.242	1.260	0.002
		Quality of chronic illness care → Health literacy → Autonomous motivation → Patient activation	0.073	0.038	0.023	0.179	0.001
		Quality of chronic illness care → Health literacy → Amotivation → Patient activation	0.073	0.042	0.014	0.191	0.011
		Quality of chronic illness care → Health literacy → Self-efficacy → Patient activation	0.098	0.054	0.024	0.249	0.005
		Quality of chronic illness care → Health literacy → Self-efficacy → Autonomous motivation → Patient activation	0.029	0.016	0.009	0.074	0.001
Health literacy	Patient activation	Health literacy → Self-efficacy → Patient activation	0.045	0.019	0.014	0.090	0.006
		Health literacy → Self-efficacy → Autonomous motivation → Patient activation	0.013	0.006	0.006	0.028	< 0.001
		Health literacy → Autonomous motivation → Patient activation	0.034	0.014	0.012	0.069	0.001
		Health literacy → Amotivation → Patient activation	0.034	0.017	0.006	0.075	0.015
		Self-efficacy → Autonomous motivation → Patient activation	0.290	0.099	0.137	0.548	< 0.001

### Multi-group analysis according to disease activity in the patient activation model 

The paths of patient activation model were examined according to disease activity. Based on K-means cluster analysis^36^, participants were classified into low disease activity group (n = 272) and high disease activity group (n = 154). And the mean of disease activity of the two groups were 3 and 11, respectively. An independent samples t-test was conducted. The results showed that the scores of PAM-13 among the low disease activity group were lower than those of the high disease activity group (t = 2.550,* p* = 0.011). The grouping was determined to be valid.

**Figure 3:**
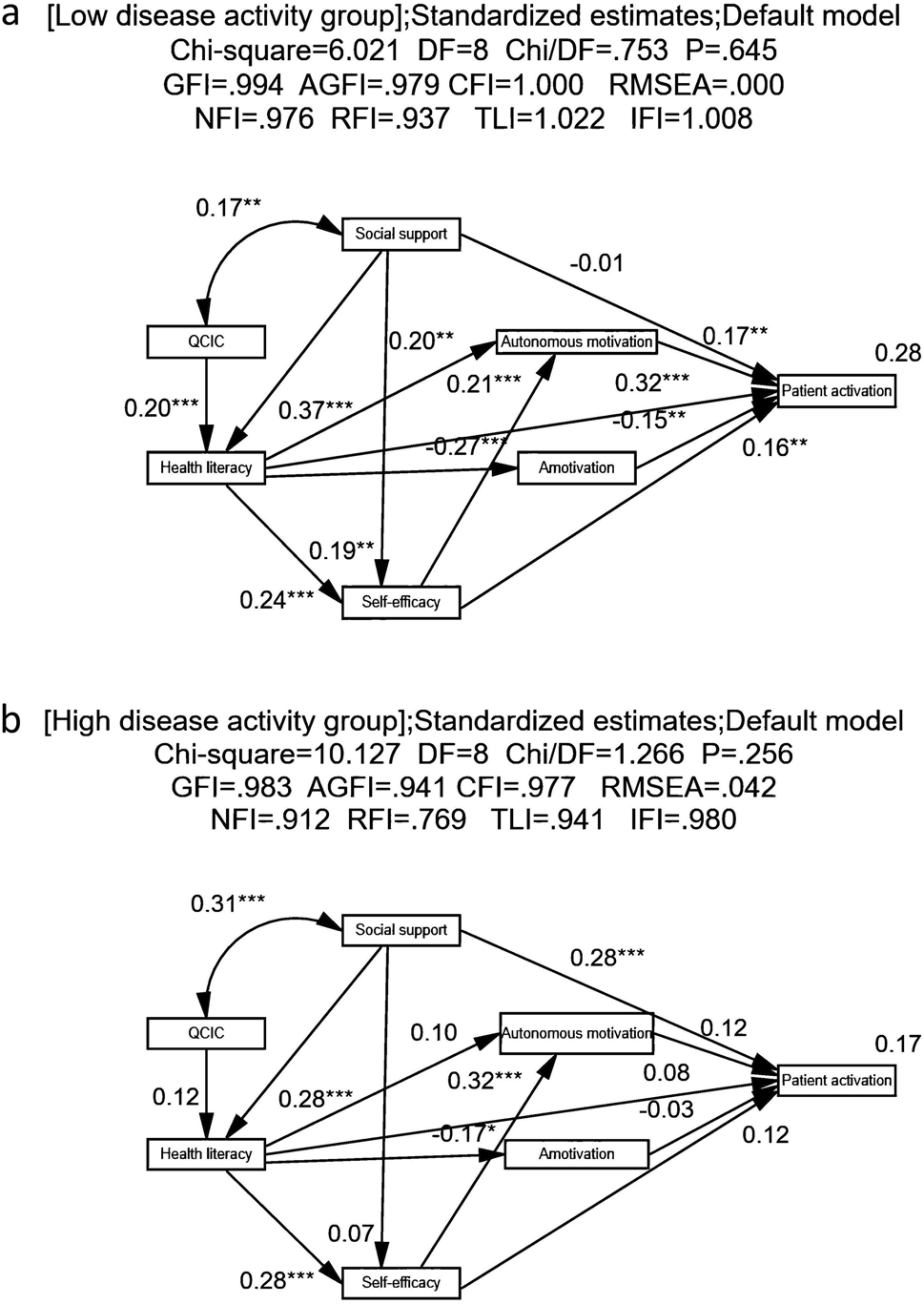
Patient activation model according to disease activity (**a**) patient activation model of low disease activity group (n = 272), (**b**) patient activation model of high disease activity group (n = 154). The coefficients for each path are standardized regression weights. QCIC, quality of chronic illness care; *p < 0.05, *p < 0.01, ***p < 0.001.

As is shown in Fig. 3, the goodness-of-fit indices for low disease activity group were χ^2^/df = 0.753, *p* = 0.645, CFI = 1.000, GFI = 0.994, AGFI = 0.979, RMSEA < 0.001, SRMR = 0.024, NFI = 0.976, TLI = 1.022, RFI = 0.937, and IFI = 1.008. The goodness-of-fit indices for high disease activity group were χ^2^/df = 1.266, *p* = 0.256, CFI = 0.977, GFI = 0.983, AGFI = 0.941, RMSEA = 0.042, SRMR = 0.047, NFI = 0.912, TLI = 0.941, RFI = 0.769, and IFI = 0.980. The goodness of fit of the models was validated to be generally good according to the decided-by-majority rule^40^.

Further analysis of a multi-group analysis of path coefficient invariance was also justified. To determine the moderating effect of disease activity on the model, both the unconstrained model and constrained model were performed^37^. The unconstrained model enabled all the paths to vary between low disease activity group and high disease activity group. Conversely, the constrained model restricted the path coefficients to be equal across the two groups. The results showed that differences between the groups were significant (Δχ^2^ = 25.805, *p* = 0.018). This suggested an interference effect of disease activity on the model and differences in the path coefficients between the models. Then, CRD was calculated to examine if the differences in each path across these two groups were significant. The path coefficients of the two groups would be regarded as significantly different at *p* < 0.05 (or 0.01) with CRD more than or equal to ± 1.96 (or 2.58). The results (Table 6) suggested that the positive effect of social support on patient activation was higher in patients with high disease activity than those with low disease activity. The positive effect of health literacy on patient activation was higher for patients in the low disease activity group. The hypothesis 2 was supported.

**Table 6:** Parameter estimates of variables for the patient activation model according to the disease activity groups (n = 426).

Path			low disease activity group			High disease activity group				Critical ratios for difference
			Standard estimate (*β*)	C.R	*p*		Standard estimate (*β*)	C.R	*p*	
Health literacy	⇠	Social support	0.373	6.795	< 0.001		0.282	3.521	< 0.001	− 0.612
Health literacy	⇠	Quality of chronic illness care	0.201	3.660	< 0.001		0.117	1.469	0.142	− 0.680
Self-efficacy	⇠	Health literacy	0.240	3.866	< 0.001		0.284	3.511	< 0.001	0.396
Self-efficacy	⇠	Social support	0.186	2.991	0.003		0.066	0.810	0.418	− 1.065
Autonomous motivation	⇠	Health literacy	0.197	3.249	0.001		0.104	1.322	0.186	− 0.964
Amotivation	⇠	Health literacy	− 0.272	− 4.651	< 0.001		− 0.166	− 2.085	0.037	0.996
Autonomous motivation	⇠	Self-efficacy	0.205	3.386	< 0.001		0.324	4.104	< 0.001	1.177
Patient activation	⇠	Autonomous motivation	0.166	3.033	0.002		0.117	1.472	0.141	− 0.815
Patient activation	⇠	Amotivation	− 0.147	− 2.724	0.006		− 0.030	− 0.396	0.692	1.538
Patient activation	⇠	Social support	− 0.005	− 0.081	0.935		0.281	3.598	< 0.001	2.824**
Patient activation	⇠	Health literacy	0.316	5.214	< 0.001		0.079	0.965	0.334	− 2.817**
Patient activation	⇠	Self-efficacy	0.161	2.844	0.004		0.122	1.478	0.139	− 0.687
Social support	⟷	Quality of chronic illness care	0.166	2.702	0.007		0.310	3.664	< 0.001	1.262

**p < 0.01.

## Discussion

This study investigated the patient activation among SLE patients in China. A patient activation model hypothesized by the Individual and Family Self-management Theory was constructed. The model was compared between the groups with low disease activity and high disease activity. The context and process variables such as health literacy and social support were found to influence patient activation, with the influential effect varying based on the degree of disease activity.

The patient activation of SLE patients was at level three. It indicated that SLE patients have started to practice health-promoting behaviors despite the lack of confidence and skills to adhere to such behaviors. This finding was consistent with the results of Fortin et al.^14^ Maintaining health-promoting behaviors can reduce the risk of flare and organ damage. Thus, focus should be directed at increasing their adherence to health promotion behaviors when enhancing patient activation. Besides, our study found that 26.1% of SLE patients had low activation. These participants were either unaware of their essential role in healthcare or deficient in the skills and confidence required for health promotion behaviours. Compared to patients with higher activation, those with lower activation could benefit more from interventions^41^. Thus, PAM-13 should be applied in clinical practice to identify patients with low activation. Meanwhile, healthcare professionals should pay greater attention to improving the belief, confidence, and skills of patients with low activation.

This study demonstrated that health literacy has the greatest impact on patient activation. The total effect included a direct (more than 70%) and an indirect effect. Patients with higher health literacy are more aware of the importance of health. They are equipped with the necessary knowledge and skills to engage in health-promoting behaviors, resulting in a higher level of activation in healthcare^42^. Additionally, health literacy can impact the process dimension where various factors such as beliefs, attitudes, and motivation interact^42,43^. This leads to the positive impact of health literacy on patient activation mediated by self-efficacy and motivation. SLE is incurable and prone to flare repeatedly. Patients with SLE must take drugs as advised, avoid exposure to certain substances, exercise moderately, and protect themselves from sunshine throughout their lives. Health literacy is vital for treat-to-target on account of its greatest influence on patient activation. Moreover, it’s a variable that can be changed. Thus, enhancing the health literacy may be more beneficial for increasing patient activation. Therefore, nurses should develop mixed health literacy-based interventions that are easy to understand with multiple forms such as pictures, videos, or brochures^44^.

We found that social support was the second most important factors affecting patient activation. Social support had direct and indirect effects on patient activation. The essential presence of an external support system in facilitating SLE patients' participation in healthcare was further supported by previous research^45^. SLE results in damage of organs and body image such as malar erythema, alopecia, and central obesity. SLE patients are heavily limited in employment, social participation, and life^2^. With a great support system, patients have better access to healthcare information, more effective management of negative emotions, and more engagement in social participation^46^. Therefore, they involve more in health management^46^. Furthermore, social support was found to have an indirect effect on patient activation through health literacy, self-efficacy, and/or motivation. The reason might be that social support could improve patients' health literacy by providing information and decision-making support and promoting communication with healthcare providers^19^. That strengthens their confidence and inner drive to effectively manage their disease^19^. This finding also revealed that variables in the context dimension could be influenced by those of the process dimension, thereby further enriching IFSMT. Additionally, influenced by the traditional concept, childlessness is considered as the most unfilial in China. As a result, women with SLE, who comprise the majority of SLE patients, suffer from tremendous stress due to restrictions on marriage and childbearing^47^. That leads to a great need for external support among SLE patients. Thus, healthcare professionals should provide more information and emotional support. SLE patients should be encouraged to take part in social activities actively. Besides, nurses should recommend that their families provide more support to the patient and increase their available external resources.

Self-efficacy was confirmed as an important factor influencing patient activation among SLE patients, exhibiting both direct and indirect effects. The result is consistent with previous findings^14^. In patients with SLE, long-term clinical remission could be achieved by medication in combination with non-pharmacological interventions^2^. Patients with higher self-efficacy have greater confidence in managing conditions successfully. Thus, they are more willing and likely to participate in disease management^48^. Consequently, self-efficacy can contribute to patient activation directly and indirectly through autonomous motivation. It has been proven that self-efficacy can be enhanced by encouragement and empowerment^16^. Therefore, healthcare providers should strive to address SLE patients' confidence in adopting health-promoting behaviors.

As hypothesized by the conceptual framework, autonomous motivation had a positive effect on patient activation, whereas amotivation negatively influenced patient activation. Despite the existing research, the relationship between motivation and patient activation has not been thoroughly explored^17^. Our findings confirmed that motivation mediated the effects of all the other antecedent variables on patient activation, identifying it as a critical precursor to activation. Therefore, interventions aimed at enhancing patient activation should target motivation. Furthermore, the influence of autonomous motivation on patient activation was found to be more substantial than that of amotivation, highlighting its importance in designing effective interventions.

Contrary to our initial hypothesis, we found no statistically significant positive correlations between introjected regulation, external regulation, and patient activation. It appeared that patients who were driven by extrinsic motivation, which encompassed introjected and external regulation, may exist in an intermediate state, situated between amotivation and intrinsic motivation^49^. While individuals with extrinsic motivation possessed greater autonomy than those who were amotivated, they exhibited less interest, confidence, and positivity in their healthcare compared to those with autonomous motivation^49^. This finding suggested that introjected and external regulations, despite being more autonomous than amotivation, did not significantly enhance patient activation. Thus, promoting autonomous motivation and reducing amotivation could be essential to enhance patient activation^49^.

The quality of chronic illness care, previously recognized as an important factor in predicting patient activation^50^, was found to have an indirect effect on patient activation in this study. The effect was mediated by health literacy, self-efficacy, and motivation. An earlier study highlighted the role of the quality of chronic illness care in patient self-management^51^. However, our findings suggest that the quality of chronic illness care had a limited impact on patient activation, possibly due to missing elements in care delivery, such as patient-centered goal setting or consistent follow-up care^52^. Inadequate care may prevent patients from realizing the importance of taking responsibility for health^52^. Additionally, SLE patients have been reported to lack disease knowledge, view themselves as passive recipients of care, and adhere to medical advice without active engagement^53^. Limited consultation time constrained healthcare providers’ ability to engage in meaningful interactions with their patients, despite their growing demands for patient education and counseling^54^. Thus, the improvement in quality of chronic illness care demands support from governmental and healthcare institutions.

Disease activity was evaluated as a moderating variable in the patient activation model for SLE patients. The results showed that disease activity could impact the paths of social support and health literacy to patient activation. Notably, in this study, the path coefficient of social support to patient activation was greater for patients with high disease activity compared to those with low disease activity. SLE patients with high disease activity suffer from serious multisystem symptoms and signs resulting from the active disease. Consequently, they are compelled to endure associated financial burdens and psychological distress. Their internal resources, such as knowledge and skills in disease management, may be insufficient to effectively address the challenges posed by the active disease. These patients tend to seek additional help to maintain health since external assistance can fill the gap^55^. Moreover, SLE patients with high disease activity have decreased social participation and increased demand for it^55^. Therefore, it is crucial for healthcare providers to enhance social support for patients with high disease activity, thereby augmenting their resources and aiding in disease management.

Besides, the path coefficient of health literacy to patient activation was higher in the low disease activity group. This suggested that health literacy played a more substantial role in patient activation among this group than in those with high disease activity. To sustain clinical remission, patients with low disease activity need to rely more on their health-related knowledge and skills in disease management^2^. For example, they should avoid SLE triggers, engage in regular exercise, and adhere to scheduled follow-up visits. However, there is a tendency for patients with low disease activity to engage less consistently in monitoring and self-management behaviors^14^. Consequently, enhancing health literacy should be prioritized for SLE patients who have low disease activity. The findings underscored the possibility that disease activity may moderate the influence of context and process dimension factors on patient activation^17^. This study also shed light on the mechanism underlying the relationship between disease activity and patient activation, emphasizing the potential benefits of tailoring interventions to the specific disease activity levels of SLE patients to bolster patient activation.

Health literacy and social support contributed more to patient activation among patients with low and high disease activity, respectively. The findings might have several implications for clinical practice. Future interventions aimed at improving patient activation among SLE patients should prioritize health literacy and social support and take disease activity into account. PAM-13 should be applied routinely in clinical practice in China and more efforts should be made to support SLE patients in maintaining health promotion behaviors. Both the context and process variables should be assessed prior to intervention development due to the existence of interactions among the antecedent factors of patient activation.

There were several limitations in this study. Firstly, although path analysis was used to validate the hypothesized conceptual framework proposed in this study, the cross-sectional design employed did not allow for the determination of causality between the variables examined and patient activation. Further longitudinal studies are needed to verify the findings of this study. Secondly, as a single-center study conducted at a tertiary hospital in Chengdu, China, the generalizability of the findings to other regions or countries is limited, which may restrict external validity. Additionally, the potential role of disease activity at the time of diagnosis as a factor in patient activation was not investigated, as many patients were diagnosed at local hospitals rather than at West China Hospital. Finally, factors such as clinical involvements, medications use, age at diagnosis and disease characteristics, which may influence patient activation, were not collected due to financial constraints. Future studies should explore the association between these factors with patient activation.

## Conclusions

Patient activation among SLE patients in China was identified as third level. Health literacy, social support, and self-efficacy were vital factors affecting patient activation. Notably, the influence of social support on patient activation was more pronounced in patients with higher disease activity, while health literacy had a greater impact on those with lower disease activity. These insights underscore the necessity and value of developing individually tailored strategies to enhance patient activation, which should be attuned to the individual's disease activity level.

Overall, healthcare professionals should provide support to SLE patients and help them to adhere to health-promoting behaviors. Health literacy, social support, and self-efficacy should be highlighted as core components in interventions for patient activation. When designing interventions, strategies should be tailored to enhance these factors based on disease activity level, with the ultimate goal of improving patient activation and the health outcomes of SLE patients.

## Data Availability

The datasets used and analyzed during the current study are available from the corresponding author on reasonable request.

## Abbreviations

SLE: Systemic lupus erythematosus
PAM: Patient activation measure
IFSMT: The Individual and Family Self-Management Theory
SLEDAI-2K: Systemic lupus erythematosus disease activity index 2000
PACIC: Patient assessment of chronic illness care
HeLMS: Health literacy management scale
SEMCD: Self-efficacy for managing chronic disease
TSRQ: Treatment self-regulation questionnaire
PSSS: Perceived social support scale
CFI: Comparative fit index
GFI: Goodness of fit Index
AGFI: Adjusted goodness of fit index
RMSEA: Root mean square error of approximation
SRMR: Standardized root mean square residual
NFI: Normed fit index
IFI: Incremental fit index
RFI: Relative fit index
TLI: Tucker and Lewis index
β: Standardized estimate
B: Unstandardized estimate
CR: Critical ratio
CRD: Critical ratios for differences
CI: Confidence interval
